# Retrospective Analysis of Adverse Drug Reactions From a Teaching Hospital in South India: Patterns, Severity, Causality, and Preventability

**DOI:** 10.7759/cureus.112854

**Published:** 2026-07-17

**Authors:** Sirpy Ram, Anandabaskar Nishanthi, Shanthi Manickam, Shravan Venkatraman

**Affiliations:** 1 Department of Pharmacology, Sri Manakula Vinayagar Medical College and Hospital, Puducherry, IND

**Keywords:** adverse drug reaction, antimicrobials, causality assessment, hartwig and siegel scale, pharmacovigilance

## Abstract

Background and aim: An adverse drug reaction (ADR) is a response to a medicinal product that is noxious and unintended, and that occurs at doses normally used in humans for the prophylaxis, diagnosis, or treatment of disease, or for the restoration, correction, or modification of physiological function. This study aimed to analyze ADRs retrospectively to assess the patterns, severity, seriousness, causality, and preventability of adverse drug reactions (ADRs) reported to an ADR monitoring center.

Materials and methods: All suspected ADRs reported from January 2020 to December 2024 to an ADR monitoring center at Sri Manakula Vinayagar Medical College and Hospital, Puducherry, which is a 1,121-bed hospital, were identified. The study period was from January 2025 to April 2025. A retrospective analysis was conducted of the clinical presentation, ADR seriousness, severity, causality, and preventability.

Results: Out of 139 ADRs reported, the majority (n=102 {73.3%}) occurred in patients aged 19-65 years, with a female predominance (n=84 {60.4%}). The most commonly affected body system was skin and subcutaneous tissue (n=93 {66.9%}), followed by general disorders (n=23 {16.5%}). The most commonly reported causal drug classes were antimicrobials (n=79 {56.8%}), followed by non-steroidal anti-inflammatory drugs (NSAIDs) (n=11 {7.9%}) and vitamin supplements (n=11 {7.9%}). Causality assessment showed (n=2 {1.4%}) certain, (n=127 {91.4%}) probable, and (n=10 {7.2%}) possible. Severity assessment showed the majority (n=124 {89.2%}) of reported ADRs were moderate, followed by mild (n=9 {6.5%}). The majority of ADRs were non-preventable (n=135 {97.1%}), followed by (n=4 {2.9%}) being definitely preventable.

Conclusion: Antimicrobials contributed to the majority of ADRs, with most reactions being probable in causality, moderate in severity, and largely non-preventable. Skin and subcutaneous tissue were the most frequently affected organ systems. Under-reporting of ADRs is suspected in our study. Our study highlights the need for vigilant monitoring during antimicrobial therapy and strengthening pharmacovigilance practices to facilitate early detection, appropriate management, and reporting of ADRs, thereby enhancing patient safety.

## Introduction

Adverse drug reaction (ADR) is defined by the World Health Organization (WHO) as “a response to a medicine which is noxious and unintended, and which occurs at doses normally used in humans” [1]. ADRs represent a significant cause of morbidity associated with medication use and can lead to or prolong hospitalization, thus constituting a significant economic burden to the healthcare system. A systematic review on the incidence of ADRs in hospitals reported that 6.34% (IQR: 3.36-16.37%) of the hospitalized patients experience ADRs, highlighting the considerable burden of ADRs in clinical practice [2].

Although clinical trials evaluate the efficacy and safety of drugs, many rare and delayed ADRs may not be detected because of limited sample size, restricted patient populations, and shorter study durations. This necessitates the need for monitoring drug safety even after marketing through pharmacovigilance. Pharmacovigilance is defined by the WHO as “the science and activities relating to the detection, assessment, understanding, and prevention of adverse effects or any other medicine/vaccine-related problem” [3]. To facilitate this, many countries have established pharmacovigilance systems, such as spontaneous reporting systems (SRS), electronic health records, patient registries, and data mining approaches to collect and analyze adverse drug events [4-7].

In India, pharmacovigilance activities are coordinated through the Pharmacovigilance Programme of India (PvPI), which operates through a network of ADR Monitoring Centers (AMCs) located in medical colleges and hospitals across the country. These AMCs play a crucial role in collecting and reporting ADRs to the National Coordination Centre (NCC). According to the performance report of the PvPI, the number of Individual Case Safety Reports (ICSRs) reported to the NCC has increased from 63,384 ICSRs in 2019-20 to 1,31,301 ICSRs in 2024-25, reflecting improved awareness and strengthening of ADR reporting systems rather than a true increase in incidence of ADRs [8].

Although some studies have been conducted to assess the occurrence and severity of adverse drug reactions across various parts of India, there is a lack of studies that provide a comprehensive assessment of ADR patterns, including causality, severity, and preventability assessments over a longer time duration [6,7]. Also, the pattern, severity, and preventability of ADRs vary across different healthcare settings and patient populations. Thus, continuous monitoring of ADRs at the institutional level is required to generate local drug safety data and strengthen pharmacovigilance practices. Hence, this study was designed to analyze the patterns, severity, seriousness, causality, and preventability of spontaneous adverse drug reactions reported to a registered ADR Monitoring Center at a tertiary care teaching hospital in South India from 2020 to 2024.

## Materials and methods

This retrospective observational record-based study was carried out in the Department of Pharmacology at a tertiary care teaching hospital, which is an approved ADR Monitoring Centre (AMC), under the Pharmacovigilance Program of India (PvPI), in South India. The ADRs reported to the AMC are periodically sent to the National Coordination Centre (NCC) through the VigiFlow software (Uppsala, Sweden: Uppsala Monitoring Centre {UMC}). The study period spanned from January 2025 to April 2025.

Study site description

The study was conducted at Sri Manakula Vinayagar Medical College and Hospital, Puducherry, a 1,121-bed private tertiary care hospital providing both inpatient and outpatient services across various specialty and super-specialty departments. The average daily outpatient consultations in 2022-2024 were 2,000-2,400, and the average daily inpatient consultations were 220-260. However, for the years 2020 and 2021, the average daily outpatient consultations were only 700 to 1,400, and inpatient admissions were 65-100 owing to the COVID-19 pandemic.

Procedure

Suspected ADR reports received at the AMC on the suspected ADR reporting forms in the data collection period from January 2020 to December 2024 were included in this study. Incomplete ADR reports were excluded from the study. The study was initiated after obtaining approval from the Institutional Ethics Committee (#EC/163/2024). Waiver of consent was granted by the Institutional Ethics Committee. Confidentiality of the participants’ data was maintained throughout the study. Data were collected from the ADR reports using a self-developed validated data collection form.

The data collection form included the year and month of ADR reporting, demographic data (age and gender), suspected drug(s) and drug class, route of drug administration, reported ADR, system organ class, ADR seriousness, causality assessment, severity assessment, and preventability assessment.

Causality Assessment

The causality of reported ADRs was categorized into certain, probable, possible, and unlikely using the WHO causality assessment scale [9]. This scale was used to assess the causal relationship between the suspected drug and ADRs reported. Causality was assessed by the causality assessment subcommittee of our institute's pharmacovigilance committee, which meets on the last working day of every month to discuss and finalize the causality of ADRs reported that month. The subcommittee includes the Head of the Department of Pharmacology (Coordinator of the PV committee), a Professor of Pharmacology (Deputy Coordinator of the PV committee), the Head of the Department of Anesthesiology, and the Chief Pharmacist; causality for each ADR is determined by consensus of the subcommittee members after discussion. It is done based on WHO-UMC causality assessment. The causality for ADR is also reported in the VigiFlow software to the National Coordination Centre of PvPI, and it is also discussed in the Quality Committee meeting held monthly.

Seriousness of ADR

The serious reactions were characterized as “death, life-threatening, increased duration of hospitalization, disability, congenital anomaly, and required intervention to prevent permanent impairment” [10].

Severity Assessment

The severity of ADRs was assessed using the Hartwig and Siegel scale, and severity of ADRs was classified into mild, moderate, and severe [11].

Preventability Assessment

The preventability of ADRs was assessed with the help of the Schumock and Thornton scale. It has three sections, namely definitely preventable, probably preventable, and non-preventable [12]. Severity and preventability assessment was independently performed by the first author (postgraduate in the department of pharmacology), the second author (professor of pharmacology and deputy coordinator of the pharmacovigilance committee), and the fourth author (assistant professor). Any discrepancies in assessment were resolved through discussion and consensus in consultation with the third author (professor and head of the department of pharmacology and coordinator of the pharmacovigilance committee).

Statistical analysis

The data were entered in Microsoft Excel (Redmond, WA: Microsoft Corp.) and analyzed using SPSS version 24 (Armonk, NY: IBM Corp.). The categorical data were expressed in terms of frequencies and percentages.

## Results

**Figure 1:**
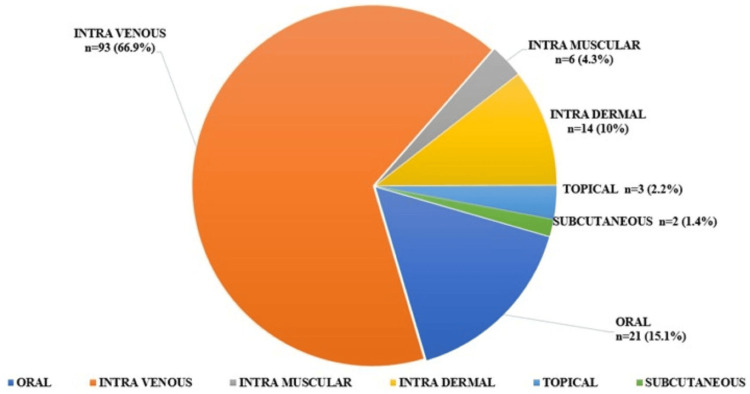
Routes of drug administration in the reported ADRs (n=139).

A total of 139 ADRs were analyzed in this study. Of the reported ADRs, the majority were observed in the age group of 19-65 years (n=102 {73.3%}), followed by individuals aged 18 years or below (n=26 {18.7%}) and those aged 65 years or above (n=11 {8%}). The maximum number of ADRs occurred in females (n=84 {60.4%}) compared to males (n=55 {39.6%}). Concomitant drug history and drug allergy were reported each in 3.6% (n=5) (Table 1). As depicted in Figure 1, intravenous route (n=93 {66.9%}) accounted for the majority of ADR reports, followed by oral route (n=21 {15.1%}). Only a few cases were associated with other parenteral (n=22 {15.8%}) and topical routes (n=3 {2.2%}).

**Table 1:** Age and gender distribution of reported adverse drug reactions (n=139). ^*^Five concomitant drugs include levothyroxine, ephedrine, ceftriaxone, ketorolac, and ondansetron.

Parameters	Frequency (%)
Age groups (years)	
≤18	26 (18.7)
19-65	102 (73.3)
>65	11 (8)
Gender	
Male	55 (39.6)
Female	84 (60.4)
Presence of concomitant drug*	5 (3.6)
History of drug allergy	
Pantoprazole	2 (1.4)
Paracetamol	1 (0.7)
Doxycycline	1 (0.7)
Unknown	1 (0.7)

**Figure 2:**
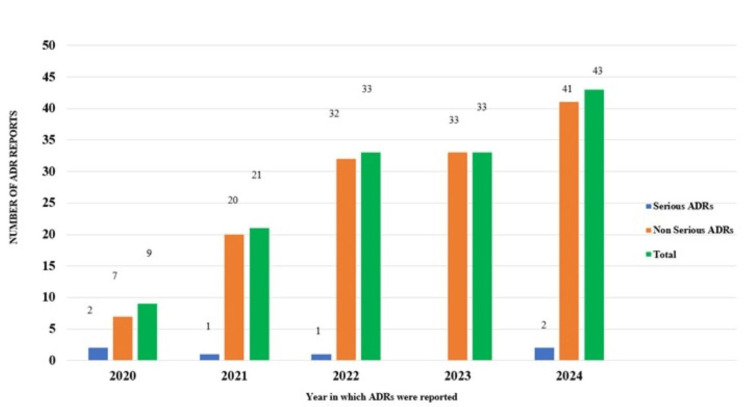
Year-wise distribution of reported ADRs based on seriousness (n=139). ADRs: adverse drug reactions

Figure 2 shows the year-wise distribution of ADR reports from 2020 to 2024. A steady upward trend in ADR reporting was observed across the years. The reporting frequency was lowest in 2020 and gradually increased from 2021 onwards, peaking in 2024. This consistent rise signifies growing awareness and active participation of healthcare providers in ADR monitoring and reporting, indicating successful implementation of pharmacovigilance programs at the institution. Serious ADRs accounted for 4.3% (n=6) of the 139 ADRs, including Stevens-Johnson syndrome (n=1), drug reaction with eosinophilia and systemic symptoms (DRESS) syndrome (n=1), angioedema (n=1), and anaphylaxis (n=3).

**Figure 3:**
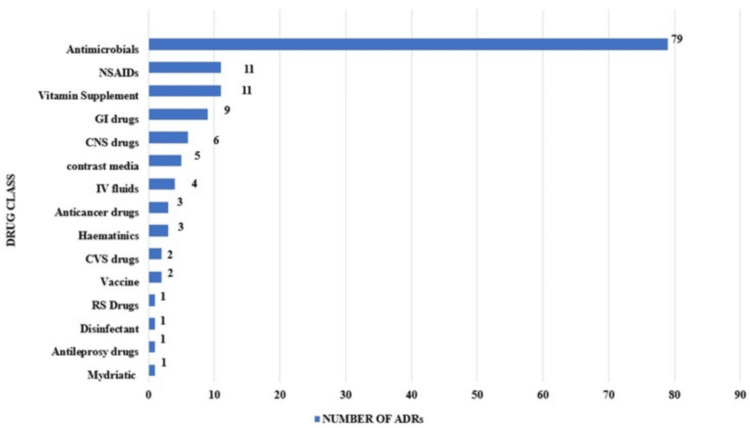
Distribution of reported adverse drug reactions (ADRs) according to drug class (n=139). NSAIDs: non-steroidal anti-inflammatory drugs; GI: gastrointestinal; CVS: cardiovascular system; RS: respiratory system

**Figure 4:**
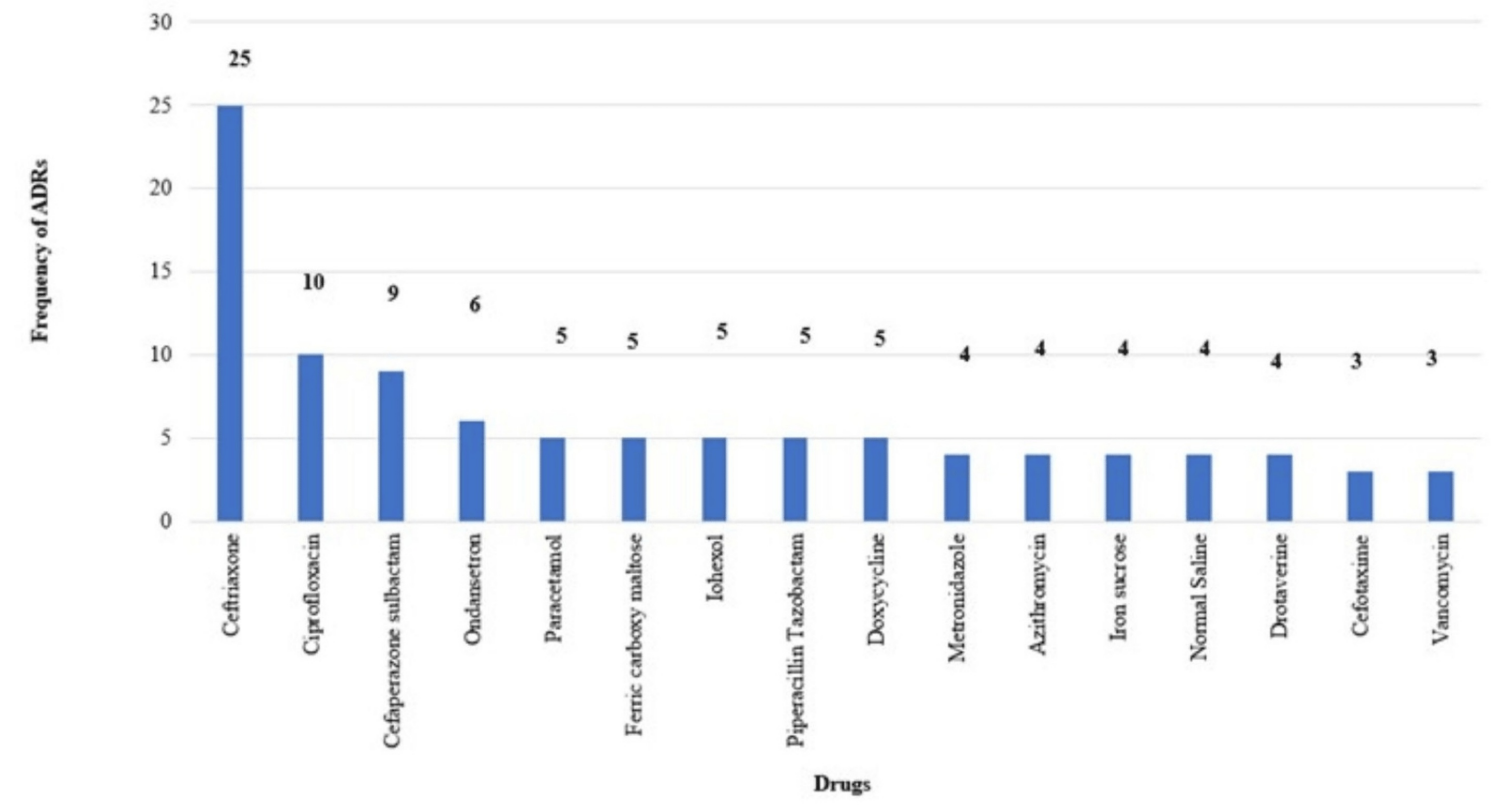
Most frequently implicated drugs in the reported adverse drug reactions (ADRs). Only drugs with ≥3 ADR reports are displayed for clarity; the remaining drugs with fewer reports are not shown.

As shown in Figure 3, the most frequently implicated drug classes were antimicrobials (n=79 {56.8%}), followed by non-steroidal anti-inflammatory drugs (NSAIDs) (n=11 {7.9%}), vitamin supplements (n=11 {7.9%}), gastrointestinal drugs (n=9 {6.5%}), and CNS drugs (n=6 {4.3%}). The most frequently implicated drugs in the reported ADRs are shown in Figure 4. Only drugs with ≥3 ADR reports are displayed for clarity, accounting for 101 of the total 139 ADRs. The remaining 38 ADRs were associated with several other drugs, each contributing one or two reports and therefore not included in the figure due to their low frequency. Figure 4 shows that ceftriaxone (n=25 {17.9%}) contributed to the maximum number of ADR reports, followed by ciprofloxacin (n=10 {7.2%}).

In terms of clinical presentation, skin and subcutaneous tissue disorders were predominant (n=93 {66.9%}), with urticaria being the most frequently reported symptom (n=80 {57.6%}). General symptoms such as generalized edema, fever, chills with rigor, and shivering accounted for 16.5% (n=23 {16.5%}), while gastrointestinal manifestations were comparatively less common (n=8 {5.8%}) (Table 2).

**Table 2:** Distribution of reported ADRs according to system organ class (n=139). SJS: Stevens-Johnson syndrome; DRESS: drug reaction with eosinophilia and systemic symptoms; ADRs: adverse drug reactions

S. no.	System organ class	ADR reports, n (%)	ADRs (n)
1	Skin and subcutaneous tissue disorders	93 (66.9)	Urticaria (80), rash (5), skin induration (4), skin discoloration (1), SJS (1), DRESS (1), angioedema (1)
2	General disorders	23 (16.5)	Generalized edema (10), fever (5), chills with rigor (3), shivering (5)
3	Gastrointestinal disorders	8 (5.8)	Vomiting (7), gum hypertrophy (1)
4	Respiratory disorders	4 (2.9)	Breathlessness (4)
5	Cardiac disorders	4 (2.9)	Palpitation (1), chest pain (3)
6	Immune system disorders	3 (2.1)	Anaphylactic reactions (3)
7	Nervous system disorders	3 (2.1)	Giddiness (3)
8	Eye disorders	1 (0.7)	Redness and watery eye (1)

The reported adverse drug reactions (ADRs) and their suspected drugs are shown in Table 3. Ceftriaxone, ciprofloxacin, and ondansetron were the most commonly implicated drugs causing urticaria, with ceftriaxone accounting for 16 of the 80 reported cases. Serious ADRs were observed with phenytoin (Stevens-Johnson syndrome), dapsone-clofazimine-rifampicin (drug reaction with eosinophilia and systemic symptoms), azithromycin (angioedema), and labetalol, cefoperazone, and ceftriaxone, each causing anaphylactic reactions.

**Table 3:** Reported ADRs and their suspected drugs (n=139). *Drugs causing urticaria with a frequency of at least three reports have been mentioned. SJS: Stevens-Johnson syndrome; DRESS: drug reaction with eosinophilia and systemic symptoms; ADRs: adverse drug reactions

S. no.	ADR (n)	Suspected drug (n)
1	Urticaria* (80)	Ceftriaxone (16), ciprofloxacin (10), ondansetron (5), drotaverine (4), piperacillin-tazobactam (4), paracetamol (4), ferric carboxymaltose (4), cefoperazone (3), vancomycin (3), cefotaxime (3), iron sucrose (3), doxycycline (3)
2	Edema (10)	Azithromycin (2), ceftriaxone (2), pantoprazole (2), calcium (2), paracetamol (1), piracetam (1)
3	Vomiting (7)	Metronidazole (2), ceftriaxone (1), tramadol (1), piperacillin-tazobactam (1), fresh frozen plasma (1), ferric carboxymaltose (1)
4	Rash (5)	Cefoperazone (2), ondansetron (1), doxycycline (1), labetalol (1)
5	Shivering (5)	Normal saline (2), metronidazole (2), iron sucrose (1)
6	Fever (5)	Normal saline (2), iohexol (1), doxycycline (1), carboplatin (1)
7	Breathlessness (4)	Cefoperazone (2), ceftriaxone (1), furosemide (1)
8	Skin induration (4)	Ceftriaxone (1), azithromycin (1), levofloxacin (1), surgical spirit (1)
9	Anaphylactic reactions (3)	Labetalol (1), cefoperazone (1), ceftriaxone (1)
10	Chills and rigors (3)	Iohexol (3)
11	Giddiness (3)	Iohexol (1), doxofylline (1), meropenem (1)
12	Chest pain (3)	Ceftriaxone (2), cefoperazone (1)
13	Skin discoloration (1)	Cefuroxime (1)
14	SJS (1)	Phenytoin (1)
15	Palpitation (1)	Ceftriaxone (1)
16	Redness and watery eye (1)	Tropicamide (1)
17	Gum hypertrophy (1)	Phenytoin (1)
18	DRESS (1)	Dapsone-clofazimine-rifampicin (1)
19	Angioedema (1)	Azithromycin (1)

The reported serious ADRs, including Stevens-Johnson syndrome (SJS), drug reaction with eosinophilia and systemic symptoms (DRESS), angioedema and anaphylaxis, were managed by immediate withdrawal of the suspected offending drug along with appropriate medical treatment, including systemic corticosteroids, antihistamines, antibiotics where indicated, and supportive care. All patients, including those with serious ADRs, showed clinical improvement and recovered completely without long-term complications during the hospital follow-up period.

Causality assessment using the WHO scale revealed that (n=127) of ADRs were “probable,” 7.2% (n=10) were “possible,” and 1.4% (n=2) were “certain.” The majority of ADRs were classified as moderate in severity (n=124 {89.2%}), while mild reactions constituted 6.5% (n=9). Six ADRs (4.3%) were classified as severe according to the Hartwig scale. Preventability assessment using the Schumock and Thornton scale indicated that most ADRs were “non-preventable” (n=135 {97.1%}) and only a few were “definitely preventable” (n=4 {2.9%}) (Table 4).

**Table 4:** Assessment of causality, severity, and preventability of ADRs (n=139). ADRs: adverse drug reactions

WHO causality assessment, n (%)	Severity assessment (Hartwig scale), n (%)	Preventability assessment (Schumock and Thornton scale), n (%)
Certain - 2 (1.4)	Mild - 9 (6.5)	Definitely preventable - 4 (2.9)
Probable - 127 (91.4)	Moderate - 124 (89.2)	Probably preventable - 0
Possible - 10 (7.2)	Severe - 6 (4.3)	Non-preventable - 135 (97.1)

## Discussion

The present study evaluated the patterns of ADRs reported to an ADR Monitoring Center at a tertiary care hospital over a five-year period, along with assessments of their severity, seriousness, causality, and preventability. Only 139 ADRs were reported during the five-year study period, suggesting possible under-reporting of ADRs. One reason was the smaller number of consultations in outpatient (OP) and inpatient (IP) settings during the COVID-19 pandemic (2020 and 2021). Also, under-reporting remains a major limitation of spontaneous reporting systems and has been widely documented in pharmacovigilance systems globally and in India [13,14]. Possible reasons for under-reporting among healthcare professionals include lack of awareness, uncertainty regarding the causal relationship between the drug and the adverse event, time constraints in busy clinical settings, fear of legal or professional consequences, and the absence of feedback or incentives for reporting.

Our study shows that the majority of ADRs occurred in adults aged 19-65 years (n=102 {73.3%}), followed by pediatric and geriatric populations. Similar findings were reported by Sharma et al. and Lobo et al., in which the adult age group accounted for the highest proportion of ADR reports due to greater exposure to pharmacotherapy [6,15]. The female predominance (n=84 {60.4%}) in this study aligns with reports from Dubrall et al. and Watson et al., suggesting that biological, hormonal, and pharmacokinetic differences may increase ADR susceptibility among females [16,17].

We found a predominance of the intravenous route (n=93 {66.9%}) implicated in ADR occurrence, which is consistent with the study by Pathak et al., who reported higher ADR frequencies with parenteral drug administration [7]. In contrast, Amin et al. reported a significant proportion of ADRs following oral drug administration [18]. These differences may be attributable to variations in study setting, patient population, and prescribing practices.

The consistent rise in ADR reporting across study years indicates a growing culture of pharmacovigilance among healthcare professionals at our hospital. In our institution, several initiatives implemented since 2021 may have contributed to the observed increase in reporting, such as regular training sessions on ADR reporting for newly recruited faculty, postgraduate students, interns, and staff nurses, as well as capacity-building programs for members of the pharmacovigilance committee to strengthen pharmacovigilance activities. In addition, Pharmacovigilance Week has been celebrated annually from September 17 to September 23 since 2021, which has helped promote awareness regarding ADR reporting among healthcare professionals. The establishment of the Quality Committee in 2021, with monthly discussions of reported ADRs, likely also promoted greater engagement in ADR monitoring and reporting. Furthermore, ongoing institutional research on strategies to improve ADR reporting among healthcare professionals and patients may have contributed to this trend. Similar trends were noted by Shin and Lee and by Vázquez-Alvarez et al., in which the implementation of structured reporting systems and real-time data monitoring significantly improved ADR surveillance [5,19].

In our study, antimicrobials were the leading drug class implicated in ADRs (n=79 {56.8%}), with ceftriaxone and ciprofloxacin being the most frequently associated agents. Similar findings have been reported by Sharma et al. and Pathak et al., in which antimicrobial agents accounted for a major proportion of ADRs in hospital-based surveillance studies [6,7]. The predominance of antimicrobial-related ADRs in our study may be explained by the high utilization of these agents in tertiary care settings, particularly for empirical therapy and management of infectious diseases. In addition, β-lactam antibiotics, such as cephalosporins, are well known for their association with hypersensitivity reactions, which may account for the higher frequency of cutaneous ADRs observed in our study. This observation is also consistent with the global pharmacovigilance data reported by Esba et al. [20].

We found that skin and subcutaneous tissue were the most frequently affected organ systems (n=93 {66.9%}), with urticaria being the predominant manifestation. These findings corroborate the results of Lobo et al. and Dubrall et al., where dermatological manifestations were among the most commonly reported ADR categories [15,16]. This underscores the importance of early dermatological evaluation in patients receiving antimicrobial therapy, as such reactions often serve as early indicators of drug intolerance.

In the present study, causality assessment using the WHO-UMC scale revealed that most ADRs were categorized as probable. However, our study findings are in contrast to the findings reported by Shukla et al., Prajapati et al., and Venkatasubbaiah et al., where possible contributed more, followed by probable [21-23]. The predominance of probable ADRs in the present study could be attributed to a clear temporal relationship between drug administration and ADR onset, along with improvement after drug withdrawal. In contrast, other studies reporting a greater proportion of possible ADRs may reflect the presence of concomitant medications or comorbid conditions that could also explain the occurrence of adverse reactions [17]. We found that only a minority (n=6 {4.3%}) of the ADRs were severe, consistent with the study by Sharma et al. in central India and Lobo et al. in Northern Brazil [6,15]. The low percentage of severe ADRs in the present study may be attributed to early recognition and timely intervention by healthcare providers, reflecting improved pharmacovigilance awareness and monitoring.

In terms of preventability assessment, 97.1% (n=135) of reactions were classified as non-preventable based on the Schumock and Thornton scale, which aligns with Kurian et al. (96.9%) [24]. This finding suggests that while most ADRs are intrinsic to drug pharmacodynamics and individual patient susceptibility, a minority could potentially be avoided through careful monitoring, rational prescribing, and dose adjustment. Furthermore, the low proportion of definitely preventable ADRs (n=4 {2.9%}) in the present study contrasts with findings of Vaman et al. (38.7%), who reported a relatively greater proportion of preventable ADRs [25]. This difference may be partly due to variations in study design and patient population, as Vaman et al.'s study was a prospective study of patients receiving antitubercular therapy for drug-resistant tuberculosis, whereas the present study was a retrospective analysis of ADRs reported to an ADR Monitoring Center over a five-year period [25]. Additionally, the lower proportion of definitely preventable ADRs in our study may indicate medication monitoring in our institution.

The limitations of this study include the possibility of under-reporting of ADRs, selective reporting, and incomplete clinical information, as the data were derived from a spontaneous reporting system. Additionally, ADR reports with incomplete information were excluded from the analysis, which could have resulted in loss of some potentially relevant cases. Furthermore, since the study was conducted in a single center, findings may not be generalizable. Future multicentric prospective studies with large sample sizes are required to obtain a more comprehensive understanding of ADR patterns and their clinical outcomes. Also, future research should explore innovative strategies to improve ADR reporting and monitoring, such as digital pharmacovigilance platforms, electronic health record integration, and patient-reported ADR systems.

## Conclusions

Antimicrobials contributed to majority of the ADRs, with most reactions being probable in causality, moderate in severity, and largely non-preventable. Skin and subcutaneous tissue were the most frequently affected organ systems. Relatively low number of ADR reports may suggest potential under-reporting. Our study highlights the need for vigilant monitoring during antimicrobial therapy and strengthening pharmacovigilance practices to facilitate early detection, appropriate management, and reporting of ADRs, thereby enhancing patient safety.

## References

[1] (2026). Safety of medicines: a guide to detecting and reporting adverse drug reactions: why health professionals need to take action. https://www.who.int/publications/i/item/WHO-EDM-QSM-2002-2.

[2] Patel TK, Patel PB (2016). Incidence of adverse drug reactions in Indian hospitals: a systematic review of prospective studies. Curr Drug Saf.

[3] (2026). What is pharmacovigilance?. https://www.who.int/teams/regulation-prequalification/regulation-and-safety/pharmacovigilance.

[4] Lee JH, Park KH, Moon HJ, Lee YW, Park JW, Hong CS (2012). Spontaneous reporting of adverse drug reactions through electronic submission from regional society healthcare professionals in Korea. Yonsei Med J.

[5] Shin H, Lee S (2021). An OMOP-CDM based pharmacovigilance data-processing pipeline (PDP) providing active surveillance for ADR signal detection from real-world data sources. BMC Med Inform Decis Mak.

[6] Sharma M, Baghel R, Thakur S, Adwal S (2021). Surveillance of adverse drug reactions at an adverse drug reaction monitoring centre in Central India: a 7-year surveillance study. BMJ Open.

[7] Pathak AK, Kumar M, Dokania S, Mohan L, Dikshit H (2016). A retrospective analysis of reporting of adverse drug reactions in a tertiary care teaching hospital: one year survey. J Clin Diagn Res.

[8] (2026). Indian Pharmacopoeia Commission: annual performance report. https://www.ipc.gov.in/mandates/pvpi/publications/8-category-en/646-annual-performance-report.html.

[9] (2026). The use of the WHO-UMC system for standardised case causality assessment. https://www.who.int/docs/default-source/medicines/pharmacovigilance/whocausality-assessment.pdf.

[10] Fatmi SM, Bagati Bagati, KD KD, Dutta S, Sharma J (2022). Characterisation of seriousness and outcome of adverse drug reactions in patients received cancer chemotherapy drugs - a prospective observational study. Curr Med Res Pract.

[11] Hartwig SC, Siegel J, Schneider PJ (1992). Preventability and severity assessment in reporting adverse drug reactions. Am J Hosp Pharm.

[12] Schumock GT, Thornton JP (1992). Focusing on the preventability of adverse drug reactions. Hosp Pharm.

[13] García-Abeijon P, Costa C, Taracido M, Herdeiro MT, Torre C, Figueiras A (2023). Factors associated with underreporting of adverse drug reactions by health care professionals: a systematic review update. Drug Saf.

[14] Tandon VR, Mahajan V, Khajuria V, Gillani Z (2015). Under-reporting of adverse drug reactions: a challenge for pharmacovigilance in India. Indian J Pharmacol.

[15] Lobo MG, Pinheiro SM, Castro JG, Momenté VG, Pranchevicius MC (2013). Adverse drug reaction monitoring: support for pharmacovigilance at a tertiary care hospital in Northern Brazil. BMC Pharmacol Toxicol.

[16] Dubrall D, Schmid M, Alešik E, Paeschke N, Stingl J, Sachs B (2018). Frequent adverse drug reactions, and medication groups under suspicion: a descriptive analysis based on spontaneous reports to the German Federal Institute for Drugs and Medical Devices from 1978-2016. Dtsch Arztebl Int.

[17] Watson S, Caster O, Rochon PA, den Ruijter H (2019). Reported adverse drug reactions in women and men: aggregated evidence from globally collected individual case reports during half a century. EClinicalMedicine.

[18] Amin S, Shah S, Desai M, Shah A, Maheriya KM (2018). An analysis of adverse drug reactions in extremes of age group at tertiary care teaching hospital. Perspect Clin Res.

[19] Vázquez-Alvarez AO, Brennan-Bourdon LM, Rincón-Sánchez AR, Islas-Carbajal MC, Huerta-Olvera SG (2017). Improved drug safety through intensive pharmacovigilance in hospitalized pediatric patients. BMC Pharmacol Toxicol.

[20] Esba LC, Al Mardawi G, AlJasser MI, Aljohani B, Alburak AA (2021). Adverse drug reactions spontaneously reported at a tertiary care hospital and preventable measures implemented. J Clin Pharm Ther.

[21] Shukla AK, Jhaj R, Misra S, Ahmed SN, Nanda M, Chaudhary D (2021). Agreement between WHO-UMC causality scale and the Naranjo algorithm for causality assessment of adverse drug reactions. J Family Med Prim Care.

[22] Prajapati K, Desai M, Shah S, Panchal J, Kapadia J, Dikshit R (2016). An analysis of serious adverse drug reactions at a tertiary care teaching hospital. Perspect Clin Res.

[23] Venkatasubbaiah M, Dwarakanadha RP, Satyanarayana SV (2018). Analysis and reporting of adverse drug reactions at a tertiary care teaching hospital. Alexandria J Med.

[24] Kurian J, Mathew J, Sowjanya K, Chaitanya KR, Ramesh M, Sebastian J, Narayanappa D (2016). Adverse drug reactions in hospitalized pediatric patients: a prospective observational study. Indian J Pediatr.

[25] Vaman RS, Thomas GD, Kalyanasundaram M, Soman S, Valamparampil MJ, Susheela RP, Murhekar MV (2025). Adverse drug reactions in persons initiated on treatment for drug-resistant tuberculosis in Kerala, India: a non-concurrent cohort study. IJID Reg.

